# Compound heterozygous mutations in a mouse model of Leber congenital amaurosis reveal the role of CCT2 in photoreceptor maintenance

**DOI:** 10.1038/s42003-024-06384-2

**Published:** 2024-06-03

**Authors:** Akiko Suga, Yuriko Minegishi, Megumi Yamamoto, Koji Ueda, Takeshi Iwata

**Affiliations:** 1 Division of Molecular and Cellular Biology, National Institute of Sensory Organs, NHO Tokyo Medical Center, Tokyo, Japan; 2 Cancer Proteomics Group, Cancer Precision Medicine Center, Japanese Foundation for Cancer Research, Tokyo, Japan

**Keywords:** Hereditary eye disease, Experimental models of disease

## Abstract

TRiC/CCT is a chaperonin complex required for the folding of cytoplasmic proteins. Although mutations in each subunit of TRiC/CCT are associated with various human neurodegenerative diseases, their impact in mammalian models has not yet been examined. A compound heterozygous mutation in *CCT2* (p.[Thr400Pro]; p.[Arg516His]) is causal for Leber congenital amaurosis. Here, we generate mice carrying each mutation and show that Arg516His (R516H) homozygosity causes photoreceptor degeneration accompanied by a significant depletion of TRiC/CCT substrate proteins in the retina. In contrast, Thr400Pro (T400P) homozygosity results in embryonic lethality, and the compound heterozygous mutant (T400P/R516H) mouse showed aberrant cone cell lamination and died 2 weeks after birth. Finally, CCDC181 is identified as a interacting protein for CCTβ protein, and its localization to photoreceptor connecting cilia is compromised in the mutant mouse. Our results demonstrate the distinct impact of each mutation in vivo and suggest a requirement for CCTβ in ciliary maintenance.

## Abstract (web summary)

The authors show that missense mutations in CCT2 evoked retinal photoreceptor degeneration with decreased expression levels of TRiC/CCT substrate proteins in the mouse retina.

## Introduction

The TRiC/CCT chaperonin complex is a eukaryotic type II chaperonin comprising eight subunits (α, β, γ, δ, ε, ζ, η, and θ) encoded by distinct genes (*TCP1*, *CCT2*, *CCT3*, *CCT4*, *CCT5*, *CCT6*, *CCT7*, and *CCT8*, respectively). TRiC/CCT was initially identified as a chaperone of actin and tubulin folding but may also assist the folding of 5%–10% of cytoplasmic proteins^1,2^ across a wide range of molecular pathways including the cell cycle, protein degradation, cellular metabolism, and G-protein-mediated signal transduction^2^. The preferred binding partners of TRiC/CCT are WD40 repeat (WDR) proteins, though the substrate specificity has not been fully elucidated^3^. The roles of TRiC/CCT have been studied in a broad range of diseases including Huntington’s disease, Parkinson’s disease, and cancers, reflecting the range of cellular processes supported by TRiC/CCT chaperone activity^4,5^. Furthermore, congenital disease studies have identified pathogenic variants in each of the TRiC/CCT subunits that are associated with distinct phenotypes, including Leber congenital amaurosis (LCA)^6^, intellectual disability^7^, Hirschsprung disease^8^, mutilating sensory neuropathy with spastic paraplegia^9^, and autism spectrum disorder^10^. However, the impacts of these variants have not been examined thoroughly in mammalian models because body-wide depletion of any TRiC/CCT subunit may affect viability.

We previously identified *CCT2* as a causal gene for autosomal recessive LCA^6^; compound heterozygous mutations in *CCT2* led to missense mutations (T400P [rs757710808] and R516H [rs762366874]) in CCTβ protein that reduced the stability of the TRiC/CCT chaperonin complex. Homozygous *Cct2* mutant zebrafish had a small eye phenotype with attenuated retinal cell differentiation resulting from a delayed cell cycle in retinal progenitor cells^11^. However, additional developmental defects (e.g., smaller head and body bent) were embryonic lethal in mutant fish. The discrepant phenotypes may result from mutation-specific differences in the zebrafish model (*cct2-L394H-7del*) and human patients (T400P and R516H) or by species-specific differences. Therefore, the effect of the LCA-causal *CCT2* mutations on the mammalian retina remains unknown.

In this report, we evaluate the pathogenicity of LCA-causal *CCT2* missense mutations (T400P and R516H) by establishing homozygous mutant mouse lines and compound heterozygotes and analyzing the resultant phenotypes. Homozygous T400P mutations evoked suspected embryonic lethality, while homozygous R516H mutants showed photoreceptor-specific degeneration starting at 4 weeks of age (4 weeks). The compound heterozygous mutations T400P and R516H affected cone cell positioning in the retina and viability in early infancy. To elucidate the proteomic changes that initiate photoreceptor degeneration, we compared protein abundance in the littermates of wild-type and homozygous R156H mutant retinas and confirmed significant decreases in TRiC/CCT substrate proteins. We also identified CCDC181, a motile cilia-associated protein, as a novel CCTβ-interacting protein. The CCDC181 protein was less abundant in the homozygous R516H mutant retina at 4 weeks and failed to localize to the connecting cilia. In contrast, in the T400P/R516H retina, CCDC181 was broadly associated with the connecting cilia, compared to its localization to the distal tip in the wild-type. Furthermore, in T400P/R516H mice, the molecule IFT88, which mediates intraflagellar transport, is also localized aberrantly around the connection point of photoreceptor cilia, indicating a functional defect in cilial transport. These observations suggest that the initiation of photoreceptor degeneration, a hallmark of the LCA phenotype in T400P and R516H *CCT2* mutants, results from intraflagellar transport dysfunction via the anomalous interaction of CCTβ with CCDC181.

## Results

### Generation of LCA model mouse with Cct2 missense mutations

**Fig. 1:**
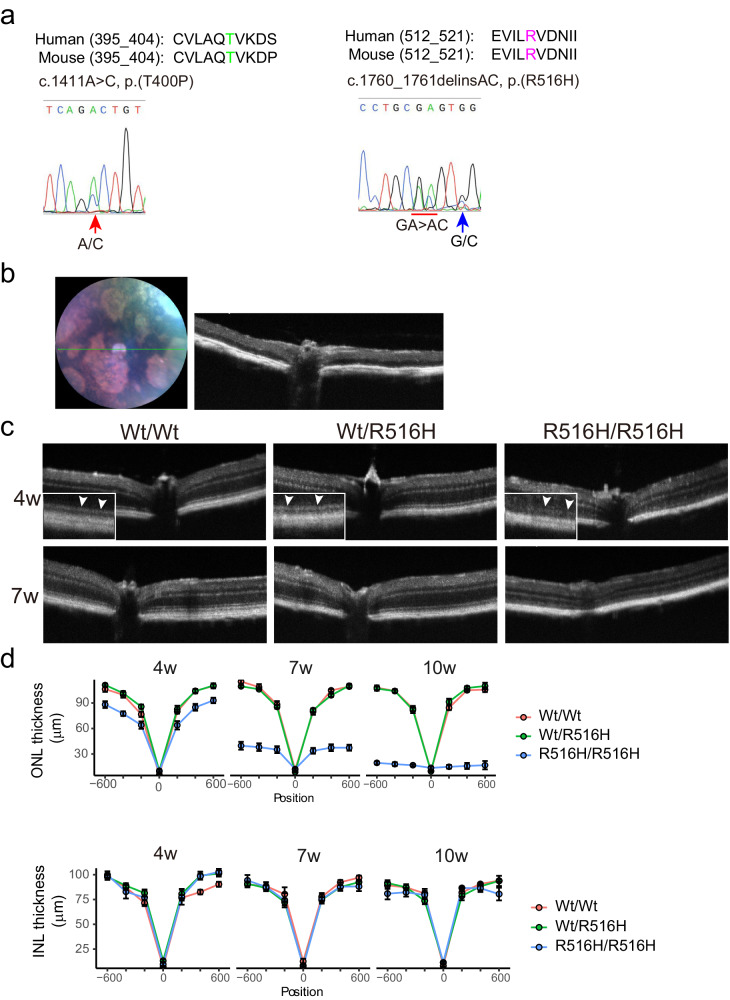
Reduced retinal thickness in R516H mutant mouse. **a** Human (NP_006422) and mouse (NP_001345696) CCTβ protein sequences around T400 (green) and R516 (magenta). Genomic DNA sequences showing the heterozygous c.1411a>c and c.1760_1761delinsAC mutations, respectively. G > C replacement (blue arrow) in R516H mutation is synonymous but makes TaqI enzyme recognition site. **b** Representative fundus and OCT images of R516H/R516H mouse at 43 weeks. **c** Representative OCT images of mice with indicated genotypes at 4 weeks and 7 weeks. Inserts shows magnified images of ellipsoid zone. **d** Quantification of ONL thickness and INL thickness from OCT images taken at the indicated time points. Distance from the optic nerve head is indicated on the x-axis (μm).

To examine the effect of two LCA-associated *CCT2* missense mutations in the mammalian retina, we generated two knock-in mouse strains carrying *Cct2* NM_007636.3:c.1411A > C p.(T400P) (hereafter, T400P) and NM_007636.3:c.1760_1761delinsAC p.(R516H) (hereafter, R516H) mutations, respectively (Fig. 1a). Mutations were confirmed by restriction digests of PCR products from mutant mice (Fig. S1a). After five cycles of heterozygous-heterozygous mating, no homozygousT400P mice were obtained (Wt/Wt = 2.6 ± 0.4, Wt/T400P = 2.0 ± 1.0, T400P/T400P = 0, p = 5.3 × 10^−4^ by chi-square test), which deviates significantly from the theoretical Mendelian ratio (Wt/Wt:Wt/T400P:T400P/T400P = 1:2:1) (Fig. S1b). Homozygous T400P mutants were not detected at embryonic day 17, meaning that homozygous T400P mutations are likely embryonic lethal. For other genotypes, litter counts fit the expected Mendelian ratios in the R516H heterozygous-heterozygous mating (Fig. S1c, Wt/Wt = 1.7 ± 0.4, Wt/R516H = 3.1 ± 0.5, R516H/R516H = 1.5 ± 0.3, p = 8.9 × 10^−1^; 17 cycles) and in the T400P heterozygous and R516H heterozygous mating (Fig. S1d, Wt/Wt = 1.6 ± 0.4, Wt/T400P = 1.2 ± 0.3, Wt/R516H = 1.7 ± 0.3, T400P/R516H = 0.9 ± 0.3, p = 1.2 × 10^−1^; 19 cycles). However, the T400P/R516H did not survive longer than postnatal 14 days (P14). Western blots of retinal lysate from mice at 4 weeks revealed a 60% decrease in CCTβ protein in R516H/R516H mice compared to Wt/Wt and R516H heterozygous (Wt/R516H) mice (Fig. S1e, g). Retinal lysate from P14 T400P/R516H mice was also depleted in CCTβ protein (Fig. S1f, h).

### Photoreceptor degeneration in R516H/R516 mice

To examine the effect of the R516H mutation on the retina, we analyzed the retinal structure of living mice using optical coherence tomography (OCT). At 43 weeks, R516H/R516H retinas were depigmented and depleted of photoreceptors (Fig. 1b). At earlier time points, retinal pathology progressed in R516H/R516H mice from a blurred ellipsoid zone (Fig. 1c, 4 weeks, arrowheads) and reduced outer nuclear layer (ONL) thickness at 4 weeks (81.7% of Wt/Wt, Fig. 1d, upper panels) to a more substantially thinned ONL at 7 week (37.0% of Wt/Wt) and 10 weeks (17.5% of Wt/Wt); the inner nuclear layer (INL) was maintained (Fig. 1d, lower panels).

**Fig. 2:**
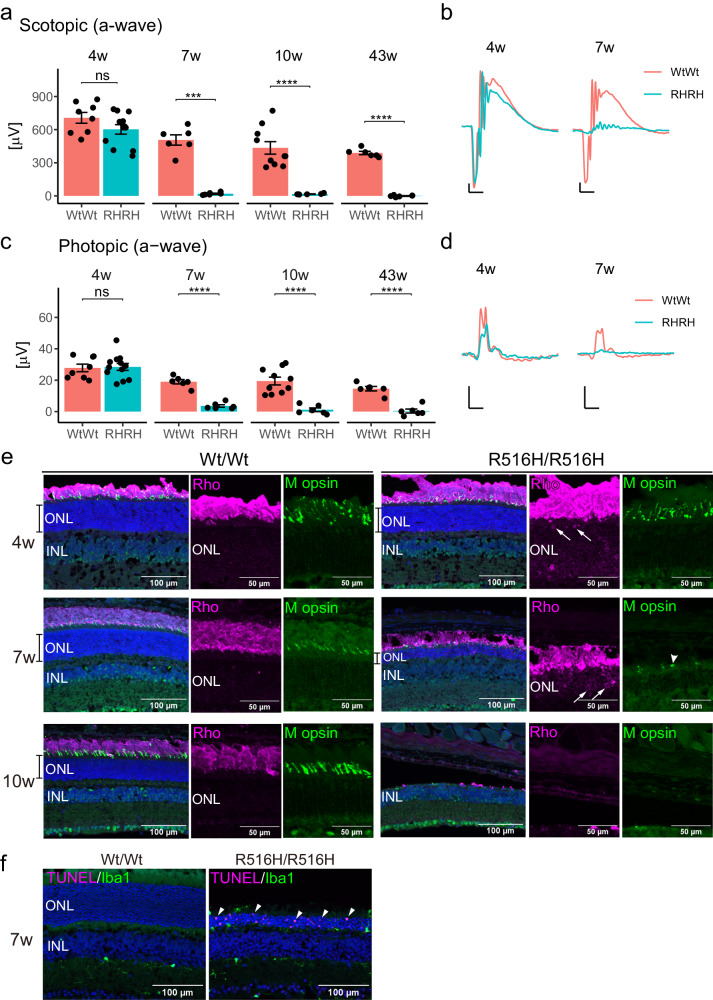
Functional and structural defects of photoreceptors in R516H mutant mouse. **a** Quantification of ERG responses from rod and cone photoreceptors at 4 weeks, 7 weeks, 10 weeks, and 43 weeks. **b** Representative scotopic ERG responses at 4 weeks and 7 weeks. **c** Quantification of ERG responses from cone photoreceptors. **d** Representative photopic ERG responses. RH/RH: R516H/R516H. For (**a**, **c**), absolute value is indicated on y-axis (mean ± SEM). Each dot indicates the value from left or right eye of the individual animal. Averaged μV is compared using *t*-test between the genotypes. ****: p < 0.0001, ***: p < 0.001, ns: not significant. Number of animals (4 weeks- n = 4 for Wt/Wt, n = 6 for R516H/R516H; 7 weeks- n = 3 for Wt/Wt, n = 3 for R516H/R516H; 10 weeks- n = 5 for Wt/Wt, n = 3 for R516H/R516H; 43 weeks- n = 3 for Wt/Wt, n = 3 for R516H/R516H). For (**b**, **d**), x-axis of the scale indicates 40 ms, and y-axis of the scale indicates 100 μV. **e** Immunohistochemical analysis of photoreceptor opsins at indicated ages. scale bars: 100 μm for marged images, 50 μm for magnified images. **f** TUNEL staining of the retinal sections from indicated genotypes at 7 weeks. scale bars: 100 μm.

Consistent with the decrease of ONL thickness during week 4 to 10, electroretinography (ERG) responses from rod and cone photoreceptors progressively decreased in R516H/R516H from week 4 to 10 (Fig. 2a–d, scotopic a-wave 4 weeks- Wt/Wt = 706.5 ± 49.1 μV, R516H/R516H = 602.4 ± 42.9 μV, p = 1.3 × 10^−1^; 7 weeks- Wt/Wt = 506.4 ± 46.6 μV, R516H/R516H = 19.4 ± 5.0 μV, p = 1.2 × 10^−4^; 10 weeks- WtWt = 434.7 ± 56.7 μV, R516H/R516H = 16.3 ± 2.3 μV, p = 4.2 × 10^−5^; photopic a-wave 4 weeks- Wt/Wt = 27.8 ± 4.1 μV, R516H/R516H = 28.5 ± 2.1 μV, p = 8.4 × 10^−1^; 7 weeks- Wt/Wt = 19.0 ± 1.4 μV, R516H/R516H = 3.6 ± 0.8 μV, p = 1.3 × 10^−6^; 10 weeks- Wt/Wt = 19.4 ± 2.5 μV, R516H/R516H = 1.2 ± 1.2 μV, p = 2.0 × 10^−5^). ERG responses were not recordable in R516H/R516H at 43 weeks (scotopic a-wave: Wt/Wt = 388.5 ± 14.8 μV, R516H/R516H = −0.94 ± 3.3 μV, p = 6.3 × 10^−7^, photopic a-wave: Wt/Wt = 14.6 ± 1.4 μV, R516H/R516H = 0.36 ± 1.3 μV, p = 2.5 × 10^−5^). Based on the significant decrease in rod and cone photoreceptor responses in R516H/R516H mice, we examined the localization of rod and cone opsin markers by immunostaining (Fig. 2e). In agreement with the OCT imaging, ONL thickness decreased during week 4–10 in R516H/R516H mouse. At 4 weeks, rhodopsin (Rho) and medium-wave-sensitive opsin 1 (M opsin) levels in the outer segment were generally not different between Wt/Wt and R516H/R516H mice. However, a small number of ONL nuclei were positive for mislocalized Rho in R516H/R516H (Fig. 2e, arrows), indicating the initiation of photoreceptor cell death. Mislocalized Rho was also detected at 7 weeks and M-opsin was detected as small puncta (Fig. 2e, arrowhead). Photoreceptor cell death during ONL thinning was confirmed by TUNEL staining in the ONL of R516H/R516H retinas at 7 weeks (Fig. 2f). These functional and histological data indicated that both rod and cone photoreceptors degenerated concurrently in the R516H/R516H retina from week 4 to 10, suggesting that rod and cone viabilities were primarily affected by R516H mutation.

**Fig. 3:**
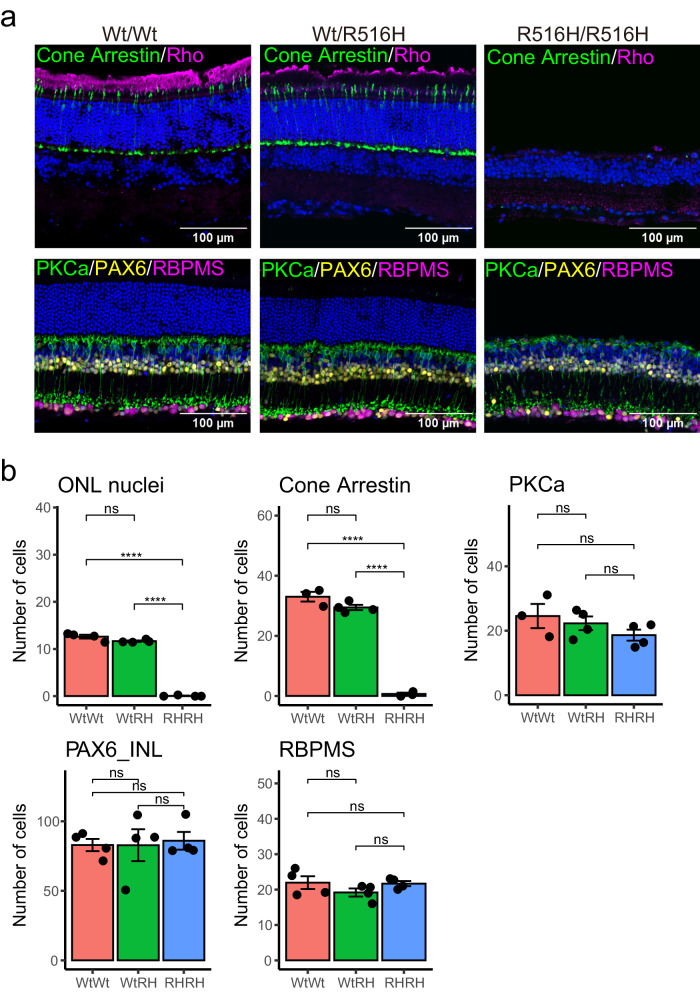
Photoreceptor-specific cell death in the R516H mutant mouse. **a** Immunohistochemical analyses of retinal sections from indicated genotypes at 10 weeks. scale bars: 100 μm. **b** Quantification of the number of cells positive for each marker (mean ± SEM). Each dot indicates the average number of cells for six sections from each mouse. Mean cell number for each genotype was compared by ANOVA with Tukey-Kramer. Wt/RH: Wt/R516H, RH/RH: R516H/R516H. ****: p < 0.0001, ns: not significant. Number of animals (n = 3 or 4 for Wt/Wt, n = 4 for Wt/R516H, n = 3 or 4 for R516H/R516H).

Because *CCT2* mRNA is widely expressed in retinal neurons with moderate expression levels in human and mouse retina (Fig. S2a, b)^12,13^, we examined the effect of R516H mutation on other retinal cell types, by immunostaining of bipolar cells, amacrine cells, and retinal ganglion cell markers at 10 weeks. Consistent with the OCT imaging, significantly fewer nuclei were detected in a single ONL column in R516H/R516H mice at 10 weeks (Wt/Wt = 12.6 ± 0.4 rows of nuclei; Wt/R516H = 11.6 ± 0.2, p = 5.7 × 10^−2^ to Wt/Wt; R516H/R516H = 0.1 ± 0.1, p = 4.6 × 10^−7^ to Wt/Wt, p = 3.2 × 10^−7^ to Wt/R516H; Fig. 3a, b). In association with the depleted Rho and M opsin staining in R516H/R516H mice at 10 weeks (Fig. 2e and Fig. 3a, upper panels), significantly fewer arrestin-positive cone cell bodies were detected (Wt/Wt = 33.0 ± 1.6 cells; Wt/R516H = 29.5 ± 0.9, p = 1.0 × 10^−1^ to Wt/Wt; R516H/R516H = 0.7 ± 0.4, p = 3.2 × 10^−7^ to Wt/Wt, p = 4.6 × 10^−7^ to Wt/R516H). On the other hand, counts of retinal bipolar cells (PKCa: Wt/Wt = 24.6 ± 3.8 cells; Wt/R516H = 22.3 ± 2.1; R516H/R516H = 18.6 ± 1.7), amacrine cells (PAX6_INL: Wt/Wt = 82.9 ± 4.4; Wt/R516H = 82.8 ± 11.4; R516H/R516H = 86.0 ± 6.4), and retinal ganglion cells (RGCs) (RBPMS: Wt/Wt = 22.0 ± 1.8 cells; Wt/R516H = 19.2 ± 1.2; R516H/R516H = 21.7 ± 0.7) were not significantly different among the three genotypes (Fig. 3a lower panels, and b). Thus, R516H mutation induces photoreceptor-specific degeneration in the mouse retina that progresses from ages 4 to 10 weeks.

### Aberrant cone cell localization in the T400P/R516H compound heterozygous mouse retina

**Fig. 4:**
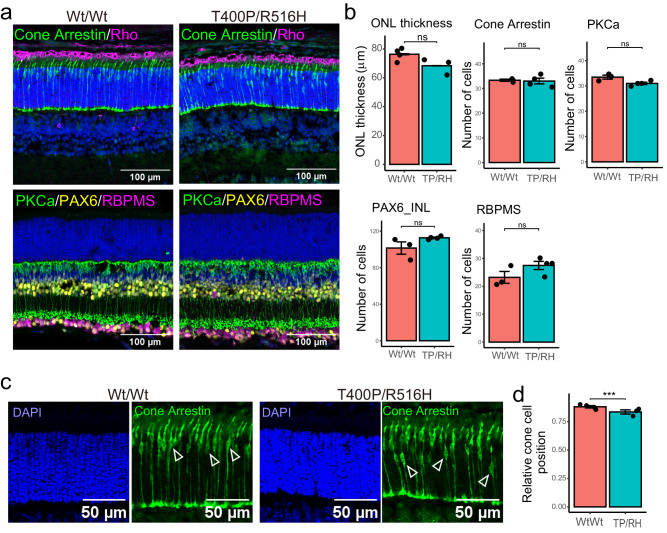
Attenuated cone cell localization in T400P/R516H mouse at P14. **a** Immunohistochemical analysis of retinal sections from indicated genotypes at P14. Scale bars: 100 μm. **b** Quantification of the ONL thickness and number of cells positive for each marker (mean ± SEM). Each dot indicates the average number of cells for six sections from each mouse. TP/RH: T400P/R516H. ns: not significant. **c** Retinal sections stained with cone arrestin and DAPI. Open triangles indicate cone cell bodies. Scale bars: 50 μm. **d** Comparison of the measured relative cone cell position between Wt/Wt and T400P/R516H (TP/RH) (mean ± SEM). Each dot indicates the mean value of relative cone cell position for each mouse. ***: p < 0.01, ns not significant. Number of animals (ONL thickness and relative cone cell position: n = 4 for Wt/Wt, n = 3 for T400P/R516H; others: n = 3 for Wt/Wt, n = 4 for T400P/R516H).

Because T400P/T400P could not be born, we could not directly examine the effect of the T400P mutation on the retina. In contrast, compound heterozygous T400P/R516H mice, which had the same genotype as previously reported patients with LCA, survived up to P14. Because depletion of TRiC/CCT function by dominant-negative PhLP1 induced photoreceptor degeneration as early as P10^14,15^, we examined if the retinal tissue structure was affected in T400P/R516H at P14 via immunostaining. Rhodopsin staining (Fig. 4a) and ONL thickness were similar in the T400P/R516H and Wt/Wt mouse retina (Wt/Wt = 76.3 ± 0.3 μm, T400P/R516H = 68.4 ± 0.1 μm, p = 1.2 × 10^−1^; Fig. 4b). Counts of cone photoreceptor cell bodies (Cone Arrestin: Wt/Wt = 33.3 ± 0.3 cells, T400P/R516H = 33.0 ± 1.2, p = 8.0 × 10^−1^), bipolar cells (PKCa: Wt/Wt = 33.5 ± 0.8, T400P/R516H = 31.0 ± 0.5, p = 6.6 × 10^−2^), amacrine cells (PAX6_INL: Wt/Wt = 101.6 ± 6.7 cells, T400P/R516H = 112.8 ± 0.8, p = 2.4 × 10^−1^) and RGCs (RBPMS: Wt/Wt = 23.2 ± 2.1 cells, T400P/R516H = 27.5 ± 1.5, p = 1.8 × 10^−1^) were similar in the Wt/Wt and T400P/R516H retinas (Fig. 4b), while some cone cell bodies were located in the middle of the ONL in T400P/R516H mice (Fig. 4c). We compared relative cone cell positions in Wt/Wt and T400P/R516H mice (pedicle to cone cell body distance divided by ONL thickness, Fig. 4d) and found that the mean relative cone cell position was slightly but significantly shorter in T400P/R516H mice (Wt/Wt = 8.8 × 10^−1^ ± 0.1, T400P/R516H = 8.4 × 10^−1^ ± 0.1, p = 6.9 × 10^−4^) (Fig. 4d), suggesting that cone cell migration was affected in T400P/R516H mice. Cone pedicle sizes did not differ significantly between Wt/Wt and T400P/R516H mice at P14 (Wt/Wt = 23.4 ± 1.3 μm^2^, T400P/R516H = 22.4 ± 2.1 μm^2^, p = 7.0 × 10^−1^). Compared to the photoreceptor-specific degeneration observed in R516H/R516H mice, more severe phenotypes were observed with the T400P/R516H (infantile death) and T400P/T400P (embryonic lethal) mutants, implying a potential dominant effect of the T400P mutation. However, Wt/T400P mice remained viable for the duration of the study (43 weeks). Further, the retinal structure in Wt/T400P mice was similar to that of Wt/Wt mice (Fig. S3a), and the scotopic and photopic responses were similar in both mouse lines at 43 weeks (Fig. S3b), suggesting that T400P is a recessive mutation in mouse as well as in the human LCA patient.

### Label-free quantification of mouse retinal proteome for LCA phenotype-associated client proteins governed by CCT2

**Fig. 5:**
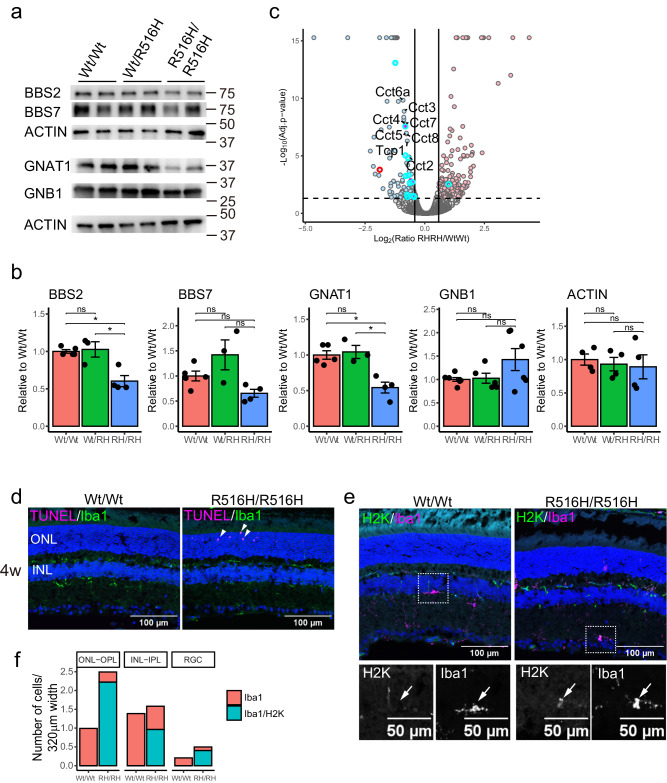
Protein expression changes in the R516H/R516H mouse retina at 4 weeks. **a** Representative image of Western blotting of retinal lysates from indicated genotype mice at 4w for BBS2, BBS7, GNAT1, GNB1 and ACTIN. **b** Quantification of retinal proteins indicated in (**a**) (mean ± SEM). Protein amount was normalized against averaged amount in Wt/Wt for each experiment. Each dot indicates the normalized protein amount for each mouse. Average protein amount for each genotype was compared by ANOVA with Tukey-Kramer. *: p < 0.01 (0.05/5), ns not significant. Data from three independent experiments were summarized. Number of animals (n = 5 for Wt/Wt, n = 3 for Wt/R516H, n = 4 for R516H/R516H). For GNB1, n = 7 for Wt/Wt, n = 5 for Wt/R516H, and n = 6 for R516H/R516H. **c** Volcano plot showing the retinal proteins differentially expressed between Wt/Wt and R516H/R516H. Proteins with significant increase in R516H/R516H are in red, significant decrease in R516H/R516H are in blue. TRiC/CCT subunits are labeled. WD40 proteins with significant change are in cyan. CCDC181 protein is colored in red. **d** TUNEL staining (magenta) of mouse retinas at 4 weeks. Microglia was labeled for Iba1 (green). **e** Immunostaining of MHC class 1 receptor (H2K, green) and microglia (Iba1, magenta) on the retinal sections from indicated genotypes at 4 weeks. **f** Quantification of the number of cells positive for Iba1 or Iba1 and H2K (Iba1/H2K) located to the indicated retinal layers.

The TRiC/CCT chaperonin complex may assist the folding of ~10% of cytosolic proteins^4^. In the retina, PhLP1 is a co-chaperone for TRiC/CCT, and conditional depletion of *PhLP1* or overexpression of FLAG-tagged dominant-negative PhLP1 in mouse photoreceptors reveals that disruption of the TRiC/CCT complex causes photoreceptor degeneration via depletion of BBS proteins and G-protein subunits in the phototransduction cascade^14,16,15^. As the R516H mutation was expected to affect TRiC/CCT chaperonin activity^6^, we examined the expression levels of known TRiC/CCT substrate proteins via western blotting for BBS2, BBS7, GNAT1, GNB1, and ACTIN in 4-weeks R516H/R516H retinas (Fig. 5a). As expected, levels of both BBS2 and GNAT1 proteins were significantly lower in the R516H/R516H mouse retina (Fig. 5b, BBS2: 60% to Wt/Wt, p = 2.7 × 10^−3^; GNAT1: 54% to Wt/Wt, p = 3.0 × 10^−3^). Levels of BBS7 protein also dropped (65% to Wt/Wt), but this result was not statistically significant (Fig. 5b). Levels of GNB1 and ACTIN did not change significantly in the R516H/R516H mutant; ACTIN levels were also unaltered in the conditional *PhLP1* knock-out retina^13^. The conditional *PhLP1* photoreceptor knock-out showed retina-wide global reductions in G-proteins including both GNAT1 and GNB1^16^, so the effects of the R516H mutation may differ from those of *PhLP1* depletion.

Based on the variable effects on the expression levels of known TRiC/CCT substrates in the R516H/R516H retina, we performed quantitative proteomic analysis to comprehensively assess the retinal proteins affected by the R516H mutation. For this experiment, retinal lysate from 4-weeks mice were used because their photoreceptors were functionally maintained and their retinal structure had not yet been remarkably degenerated. In addition, no significant change in the rod-specific protein GNB1 in the R516H/R516H retina was observed by western blotting, indicating that an adequate number of ONL cells for comparing the protein expression remained at this stage. Protein expression levels were compared in Wt/Wt and R516H/R516H mouse retinas via label-free quantitative mass spectrometry (LFQ-MS, Fig. 5c). In total, 8780 proteins were identified, of which >96.6% (8531 proteins) were shared across the Wt/Wt (8664 proteins) and R516H/R516H (8647 proteins) retinal proteomes. Quantitative protein abundance was obtained for 8482 of 8780 identified proteins. *p* < 0.05 were considered statistically significant for proteins shown in the volcano plot. The full list of identified proteins by LFQ can be found in Supplementary Data 1. We found that abundance decreased for 108 proteins in the mutant retina and increased for 137 proteins. Consistent with Western blotting results, GO analysis revealed that all eight subunits of the TRiC/CCT chaperonin complex, BBS proteins, and ciliary proteins were depleted in the R516H/R516H retina (Table 1, Fig. 5c). We further found that WDR proteins, known as preferred binding partner of TRiC/CCT^3^, were also depleted in the R516H/ R516H mouse retina (Fig. 5c, cyan circles; Table 2, p = 2.9 × 10^−3^, Fisher exact test). Again, although GNB1 has a WDR domain, levels were unchanged in the mutant retina, suggesting that WDR proteins are not all equal clients of the CCT2 chaperone. Nonetheless, these proteomics results indicate that the function of TRiC/CCT is indeed suppressed in the retina of the established *CCT2*-LCA model mouse.

**Table 1:** Top-10 list of the GO enrichment analysis

GO cellular component complete	Fold Enrichment	Raw P-value	FDR
**Downregulated proteins**			
chaperonin-containing T-complex (GO:0005832)	97.18	7.E-13	1.E-09
box H/ACA telomerase RNP complex (GO:0090661)	80.99	7.E-04	4.E-02
BBSome (GO:0034464)	75.93	4.E-08	1.E-05
zona pellucida receptor complex (GO:0002199)	74.76	3.E-12	3.E-09
chaperone complex (GO:0101031)	35.99	3.E-10	2.E-07
pericentriolar material (GO:0000242)	22.09	6.E-05	5.E-03
Cul4-RING E3 ubiquitin ligase complex (GO:0080008)	15.67	2.E-04	1.E-02
ciliary transition zone (GO:0035869)	13.13	4.E-07	8.E-05
photoreceptor outer segment (GO:0001750)	8.68	1.E-04	8.E-03
photoreceptor cell cilium (GO:0097733)	8.31	9.E-06	1.E-03
**Upregulated proteins**			
interphotoreceptor matrix (GO:0033165)	69.14	8.91E-04	4.73E-02
TAP complex (GO:0042825)	69.14	8.91E-04	4.61E-02
symbiont-containing vacuole membrane (GO:0020005)	38.89	1.34E-04	1.05E-02
host cell cytoplasm (GO:0030430)	34.57	1.77E-04	1.29E-02
symbiont-containing vacuole (GO:0020003)	34.57	1.77E-04	1.24E-02
host cellular component (GO:0018995)	34.57	1.77E-04	1.20E-02
host cell cytoplasm part (GO:0033655)	34.57	1.77E-04	1.16E-02
host intracellular part (GO:0033646)	34.57	1.77E-04	1.12E-02
host cell part (GO:0033643)	34.57	1.77E-04	1.09E-02
host cell (GO:0043657)	34.57	1.77E-04	1.06E-02

**Table 2:** Distribution of WD40 repeat proteins

	Protein groups			
RH/RH vs Wt/Wt	WD40	Not WD40	adjusted p-value	p.adj.signif
Higher	1	136	0.205	ns
No change	312	7798	0.714	ns
Lower	12	96	0.0029	**

Higher: RH/RH vs Wt/Wt > 1.5, p < 0.05; Lower: RH/RH vs Wt/Wt < 0.75, p < 0.05; multiple Fisher exact test, p-value was normalized by Bonferroni.

**significant.

Proteins with increased abundance in the R516H/R516H mouse retina are involved in genomic DNA excision repair which follows excess double-strand breaks, and MHC-Class I (TAP complex, Table 1). The Class-I human MHC molecule, human leukocyte antigen (HLA), is upregulated following DNA damage caused by irradiation in human cell lines including retinal pigment epithelium in vitro; aberrant protein translation is induced by increased pioneer-round translation, and these aberrant proteins are processed and presented for immune cell recognition as class I immunopeptides^17^. To examine whether MHC-Class I proteins were activated in retinal neurons with DNA damage, we tested the localization of TUNEL-positive cells and mouse MHC-Class I protein (H2K) by immunostaining. We detected TUNEL-positive cells in the ONL of R16H/R516H at 4 weeks that indicate double-strand breaks in photoreceptor nuclei (Fig. 5d), while MHC-Class I protein (H2K) was detected on Iba1-positive microglia in the ONL, INL, and GCL of R516H/R516H (H2K/Iba1, Fig. 5e, f). Iba1-positive microglia were more abundant in the ONL and OPL of R516H/R516H retinas than of Wt retinas (Wt/Wt = 1.0 ± 0.4, R516H/R516H = 2.5 ± 0.4, p = 2.7 × 10^−2^), and the majority of Iba1-positive microglia were also H2K-positive (89.1%, Fig. 5f). Thus, unlike retinal pigment epithelium, MHC-Class I expression was enhanced by an unclear mechanism in microglia, which was activated by the apoptotic photoreceptors in R516H/R516H mouse.

### Mislocalization of CCDC181 in the R516H/R516H and T400P/R516H mouse retina

**Fig. 6:**
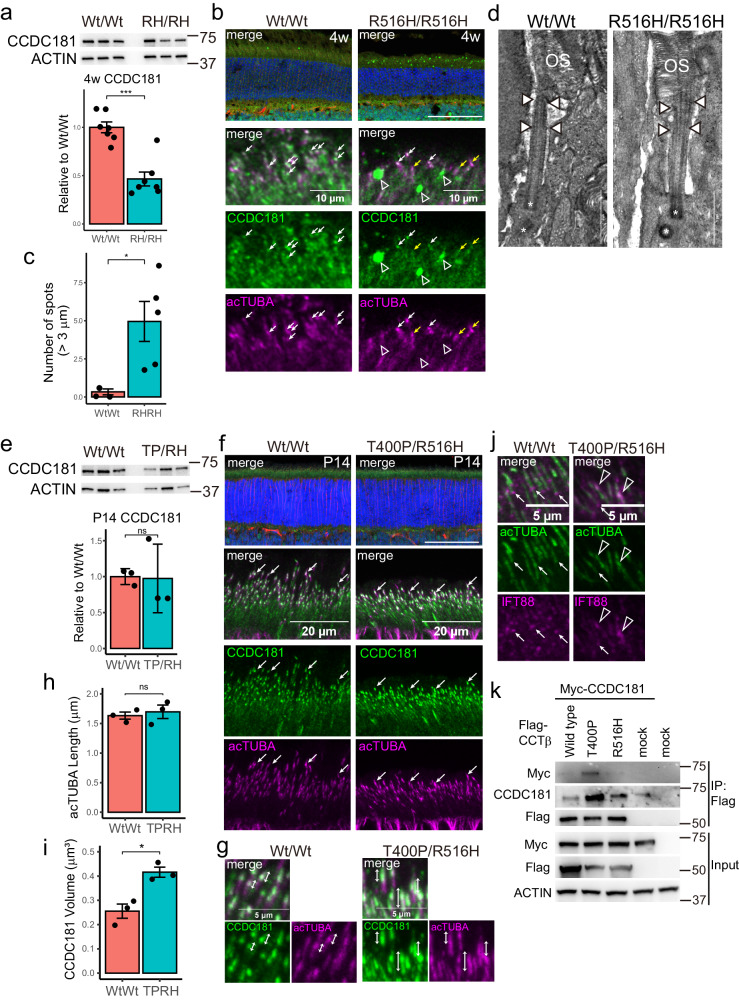
Mislocalization of CCDC181 in *Cct2* mutant retinas before photoreceptor degeneration. **a** Quantification of CCDC181 protein amount normalized to actin in the retinas from indicated genotypes at 4 weeks. Bar graph represents mean ± SEM. Each dot indicates the normalized protein amount for each mouse. **b** Immunostaining of CCDC181 on the retinal sections from indicated genotypes at 4 weeks. Arrows indicate co-localization of CCDC181 (green) and acTUBA (magenta). Open triangles indicate CCDC181-positive spots. Scale bars: 100 μm for the top panels, 10 μm for the lower panels. **c** Quantification of the number of CCDC181-positive spots larger than 3μm^2^ (mean ± SEM). Each dot indicates the number of spots averaged for six sections from each mouse. Number of animals (n = 4 for Wt/Wt, n = 5 for R516H/R516H). **d** Representative electron microscopy of the photoreceptor primary cilia at 4 weeks. White rectangles indicate the connecting cilia. OS: outer segment, asterisks indicate basal bodies. Scale bars: 1 μm. **e** Quantification of CCDC181 protein amount normalized to actin in the indicated genotypes at P 14. Bar graph represents mean ± SEM. TP/RH: T400P/R516H. Number of animals (n = 3 for Wt/Wt, n = 3 for T400P/r516H). **f** Immunostaining of CCDC181 (green) and acTUBA (magenta) on the retinal sections from indicated genotypes at P14. Stacked images are shown for the lower panels. Arrows indicate co-localization of CCDC181 and acTUBA. Scale bar: 100 μm for the top panels, 25 μm for the lower panels. **g** Magnified stacked image of connecting cilia stained for CCDC181 (green) and acTUBA (magenta). Bidirectional arrows indicate the co-localization of CCDC181 and acTUBA. **h** Quantification of connecting cilia length stained by acTUBA in stacked retinal sections from indicated genotypes (mean ± SEM). Each dot indicates the mean length for each animal. TP/RH: T400P/R516H. Number of animals (n = 3 for Wt/Wt, n = 3 for T400P/R516H). **i** Quantification of CCDC181 immunostained volume in stacked retinal sections from indicated genotypes (mean ± SEM). Each dot indicates the mean volume for each animal. TP/RH: T400P/R516H. Number of animals (n = 3 for Wt/Wt, n = 3 for T400P/R516H). **j** Immunostaining of acTUBA (green) and IFT88 (magenta) in the retinal sections from indicated genotypes. Arrows indicate IFT88 localized to the distal tip of connecting cilia. Open triangles indicate aberrant localization of IFT88 in T400P/R516H retinas (representative image for three mice for each genotype). **k** Immunoprecipitation of Flag-tagged CCTβ proteins and Myc-tagged CCDC181 in HEK293T cell lysate. Overexpressed proteins are indicated on the top. *: p < 0.05, **: p < 0.01, ***: p < 0.001, ns not significant.

To further examine the effects of the R516H mutation in photoreceptor cell death initiation, we focused on CCDC181 (Fig. 5c, red circle), a microtubule-associated protein reported to localize to motile cilia of airway epithelial cells and sperm flagella^18,19^. CCDC181 was more severely depleted than TRiC/CCT subunits and BBS components (LFQ data in Supplementary Data 1) and was the only known ciliary protein among the 10 most depleted proteins in the R516H/R516H mouse retina; the association between CCDC181 and retinal function/pathology has yet to be studied. In our study, CCDC181 overexpression in NIH3T3 cells increased the ratio of acetylated alpha-tubulin (acTUBA) to general alpha-tubulin (TUBA) after 3 days of serum starvation (Fig. S4a) and promoted the accumulation of acTUBA in the cell (Fig. S4b, c), suggesting that CCDC181 contributes to primary cilia formation. Western blotting confirmed the depletion of CCDC181 protein by 50% in the R516H/R516H retina at 4 weeks (Fig. 6a), and immunohistochemical analysis revealed that CCDC181 localized to the acTUBA-labeled primary connecting cilia of photoreceptors at 4 weeks (Fig. 6b, Wt/Wt, white arrows). However, CCDC181 immunostaining showed larger spots in the photoreceptor inner segment in the R516H/R516H retina (Fig. 5b, open triangles) that did not co-localize with acTUBA. We detected significantly more CCDC181-positive large spots (>3 μm^2^) in the R516H/R516H mouse inner segment (Wt/Wt = 0.3 ± 0.2, R516H/R516H = 5.0 ± 1.3 spots per 160 μm width, p = 1.9 × 10^−2^; Fig. 6c). In contrast to its accumulation outside the connecting cilia, we found reduced CCDC181 co-localization on the tips of the connecting cilia in the R516H/R516H retina (Fig. 6b, yellow arrows). We further examined the effect of the R516H mutation on the structure of the photoreceptor primary cilia at 4 weeks using electron microscopy (Fig. 6d). The outer segment (OS) and basal body (asterisks) appeared normal. We detected spreading microtubules in the distal part of the connecting cilium (white rectangles) in a small number of R516H/R516H mouse photoreceptors.

Although photoreceptor degeneration was not apparent in the T400P/R516H mouse retina at P14, the aberrant cone photoreceptor positioning suggests that compound heterozygous mutations affect retinal proteins to some extent. Therefore, we also tested whether CCDC181 localization in the photoreceptor was altered in T400P/R516H mice. At P14, CCDC181 expression levels were not significantly different between Wt/Wt and T400P/R516H (Fig. 6e). CCDC181 co-localized with distal tips of acTUBA-labeled connecting cilia in Wt/Wt retina (Fig. 6f arrows, and Fig. 6g bi-directional arrows), but expanded to the proximal part in T400P/R516H mouse retinas (Fig. 6g, bi-directional arrows). Although cilia lengths were not significantly different (Wt/Wt = 1.7 ± 0.3μm, T400P/R516H = 1.7 ± 0.4 μm, p = 2.9 × 10^−1^, Fig. 6h), the volume of CCDC181-positive spots did increase significantly in the T400P/R516H retina (p = 8.4 × 10^−10^, Fig. 6i), suggesting that CCDC181 protein transport was compromised in primary cilia. We also examined intraflagellar transport protein IFT88^20^ in P14 cilia. In Wt/Wt mouse photoreceptors, IFT88 accumulated to the proximal side of acTUBA-labeled connecting cilia (Fig. 6j, arrows), whereas in the T400P/R516H mouse retina, ITF88 was more localized on the intermediate or distal side of the connecting cilia (Fig. 6j, open triangles).

The localization of CCT2 at connecting cilium^6^ prompted us to examine protein interactions between CCDC181 and the CCT2 mutants. Immunoprecipitation experiments with Flag-tagged CCT2 revealed that the T400P mutant has a higher affinity for CCDC181 than the wild-type and R516H mutant variants (Fig. 6k). In summary, LCA-associated *CCT2* mutations affect ciliary protein localization prior to robust photoreceptor degeneration in mouse models.

## Discussion

In this study, we generated mice carrying T400P and R516H mutations in *CCT2* that are causal for LCA in a compound heterozygous model. Mutant mouse phenotypes ranged from embryonic lethality to photoreceptor degeneration and paralleled the impact on the TRiC/CCT complex as determined in vitro. Retinal proteomic analysis revealed a depletion of TRiC/CCT substrate proteins and mislocalization of CCDC181, a CCT2-interacting protein, at photoreceptor connecting cilia during the initiation of photoreceptor degeneration.

The T400P and R516H mutations in *CCT2* were first identified as a compound heterozygote in familial LCA patients. In general, the contribution of each individual mutation to LCA disease pathogenesis in the compound heterozygote has been overlooked and unstudied; we found no published examinations of this aspect in a compound heterozygous experimental model. In this study, we generated mouse lines carrying each mutation independently to clarify the impact of each. Contrary to accepted belief, the biallelic inheritance of each of the LCA-associated compound heterozygous *CCT2* mutations showed distinct phenotypes in vivo. The T400P homozygote was embryonic lethal (indicating the more negative impact on CCT2 protein function), the R516H homozygote exhibited photoreceptor degeneration at 4 weeks of age, and the compound heterozygote T400P/R516H was inviable past P14 with a retinal phenotype of attenuated ciliary transport in photoreceptor cells. The T400P mutant contains an alpha-helix-breaking substitution at position 400 and interacts with HSP90, unlike R516H^6^. HSP90 is a protein-refolding chaperone for misfolded proteins, and the binding of HSP90 to T400P indicates structural instability in the T400P mutant that leads to more rapid (compared to wild-type) proteasomal degradation after translation. A previous report of a truncated *CCT2* mutant zebrafish confirms the embryonic lethality and CCT2 protein depletion phenotypes^11^. The in vivo severity of each mutation was consistent with previous in vitro experiments; rapid degradation of structurally unstable T400P mutant protein resulted in more global hypofunction of TRiC/CCT similar to a knock-out that causes more severe phenotypes compared to the structurally stable but functionally defective R516H mutant protein. In contrast, the early postnatal lethality of the T400P/R516H mouse suggested species-specific susceptibility to this mutation, since human patients carrying the same mutation were clinically diagnosed with LCA at 6 months of age. Unfortunately, long-term clinical records which would improve the genotype-phenotype correlation of the *CCT2* mutations, are not available for these patients.

Proteomics comparisons of wild-type and R516H retinas revealed severe depletion of TRiC/CCT subunits and substrates in the mutant retina that indicate reduced R516H TRiC/CCT chaperonin activity, which is consistent with predictions from 3D protein structure modeling^6^. We found that the number of photoreceptor-specific proteins, such as Rho (P15409), M-opsin (O35599), RPGRIP1 (Q9EPQ2), CNGA1 (P29974), and PDE6B (P23440), was not significantly altered by LFQ (Supplementary Data 1). Maintenance of these photoreceptor-specific proteins, but a significant decrease in WDR proteins, further supported that the R516H mutation specifically affected TRiC/CCT substrate proteins. However, the R516H mutant phenotype was different from previous reports which suppressed TRiC/CCT activity in the retina. For example, LFQ and WB experiments revealed no depletion of GNB1 protein. Previous reports used conditional depletion of PhLP1^16^ or overexpression of Flag-tagged dominant-negative PhLP1^14^ to suppress TRiC/CCT chaperonin activity; PhLP1 is a co-chaperone for GNB1^21^, and previous reports may have emphasized PhLP1-mediated TRiC/CCT function in the retina. Furthermore, rapid and concurrent degeneration of rod and cone photoreceptors in R516H retina suggested that the mutation primarily affected the viability of both photoreceptors. In contrast to the rapid photoreceptor degeneration in the rod-specific PhLP1 knock-out, cone-specific depletion of PhLP1 did not cause photoreceptor degeneration, despite the significant decrease of cone-specific G-proteins^22^. These results would suggest that R516H mutation affects the PhLP1-mediated TRiC/CCT chaperonin function rather moderately, but has broad impact on proteins required for the maintenance of both photoreceptors.

GO analysis revealed a depletion of ciliary proteins in the R516H retina. In addition to the previously reported TRiC/CCT client ciliary proteins (e.g., BBS2, BBS4, and BBS7)^15^, we identified CCDC181 as depleted in the mutant retina. We found that CCDC181 enhanced alpha-tubulin acetylation in vitro, localized to the photoreceptor connecting cilia, and interacted with CCT2. The meaning of this interaction as a substrate for TRiC/CCT or a cilium-associated function has yet to be investigated. Because CCDC181-positive large spots in the R516H/R516H implied an increase of misfolded protein aggregation, we compared CCDC181 with a known TRiC/CCT substrate protein, GNAT1, by determining the soluble/insoluble ratios in retinal lysate (Fig. S5). While GNAT1 increased substantially in the insoluble fraction of the R516H retina, suggesting an increase in unfolded protein aggregates, CCDC181 did not change significantly between wild-type and R516H retinas. This result does not prove whether or not CCDC181 is a substrate of TRiC/CCT, however, it supports the variable impact of the R516H mutation on the CCTβ-interacting proteins. R516H mutation possibly affected a cilia-associated function mediated by the interaction of CCDC181 with CCTβ, similar to the interaction of p150^Glued^ with CCTδ monomer in dynein-mediated microtubule transport^23^. Considering the recently reported role of CCTβ as an aggrephagy receptor for PolyQ-huntingtin in inclusion bodies^24^, this additionally suggests a possible impact on aggrephagy in photoreceptors.

Mislocalization of the cone photoreceptor nuclei in the T400P/R516H mouse retina at P14 additionally suggests a role of CCTβ in the interkinetic nuclear migration during retinal development^25^. Linker of the nucleoskeleton to the cytoskeleton (LINC complex) is one of the mechanisms that regulate the interkinetic nuclear migration by connecting the nuclear membrane to the microtubule. Disruption of the LINC complex causes mislocalization of cone cell nuclei to the basal side at P8^26^. Thus, cone photoreceptor mislocalization and impaired ciliary transport in T400P/R516H at P14 both support the impact on microtubule-associated function of CCTβ. The role of CCTβ in CCDC181-mediated microtubule modification will soon be clarified in a future study.

In conclusion, by generating mice carrying each of the LCA-associated compound heterozygous CCT2 mutations (T400P and R516H), we demonstrate the divergent in vivo impacts of these individual missense mutations, ranging from embryonic lethality in T400P homozygotes to photoreceptor degeneration in R516H homozygotes. Photoreceptor degeneration in R516H homozygotes was associated with depletion of TRiC/CCT substrate proteins and the mislocalization of a CCTβ-interacting ciliary protein, CCDC181. These data suggest the importance of TRiC/CCT-mediated protein folding and CCTβ for the maintenance of photoreceptors.

## Methods

### Ethics

Animal experiments were conducted in accordance with the Association for Research in Vision and Ophthalmology Statement for the Use of Animals in Ophthalmic and Vision Research, and approved by the Tokyo Medical Center Experimental Animal Committee. We have complied with all relevant ethical regulations for animal use.

### Generation of Cct2 T400P and Cct2 R516H mutant mice by CRISPR/Cas9

Guide sgRNAs were designed using the GPP sgRNA designer. To generate the *Cct2* T400P mouse line, two sgRNAs (#1: 5′-GCATGCACCATTGTGCTTCG-3′; #2: 5′- GATCCTAGAACAGTTTACGG-3′) and a synthesized donor oligo with the c.1411A>C mutation were used: 5′-TGGCTAGACGGTCACTGATTGACGTTTCTCCTTAGGTGAGGCATGCACCATTGTCCTGCGTGGTGCCACTCAGCAAATTCTGGATGAAGCTGAACGATCTCTGCATGATGCTCTTTGTGTTCTTGCTCAGCCTGTAAAAGATCCTAGAACAGTATATGGGGGAGGTAAGCATTTCAAGAATGTTACATACTTTGTTTCTT-3′. The c.1411A>C replacement creates an EspI enzyme restriction site. To generate the *Cct2* R516H mouse line, a single sgRNA (5′-TGATAATGTTGTCCACTCGC-3′) and synthesized donor oligo with c.1760_1761delinsAC were used: 5′-AAGCGACAGGTTCTTCTGAGTGCGGCTGAAGCAGCAGAGGTGATCCTGCACGTCGACAACATTATCAAAGCAGCACCAAG-3′. We also used a synonymous c.1764G>C mutation to create a TaqI restriction enzyme site in the donor oligo. Microinjection, embryo transplantation, founder mouse generation, and animal care were carried out by the Institute of Immunology (Tochigi, Japan). Mice were kept under a standard 12 h light/dark cycle. Heterozygous founder mice were backcrossed to C57BL6/J mice for at least three generations. Male and female mice were mixed for statistical analysis. Ages are indicated in each figure legend.

### Genotyping of *Cct2* T400P and R516H mice

Genotyping of mice was performed at 2–3 weeks after birth. Genomic DNA was extracted from tail tips and amplified by PCR using Prime Star HS DNA Polymerase (Takara) with specific primer pairs for each mutation (*Cct2* T400P: T400P_Fw: 5′-TGCAGAGAAGCATTGCTACCAAAGCC-3′, T400P_Rv: 5′-ATGGGCCATCAGCATCTCAGAGCA-3′; *Cct2* R516H: R516H_Fw: 5′-TCTAGCTAAATCAGGCAGTGAGATGCAGT-3′, R516H_Rv: 5′-TGGCTACCCAAACCAGTTTCCTCCT-3′). To detect the T400P mutation, the PCR-amplified DNA fragment was digested with EspI restriction enzyme (Takara) and detected by electrophoresis and imaging. To detect the R516H mutation, the TaqI restriction enzyme (Takara) was used.

### OCT imaging

In vivo imaging of the retinal thickness was performed by optical coherence tomography (OCT) using micron IV (Phoenix Technology group). Mice were anesthetized by intraperitoneal injection of a combination anesthetic (0.3 mg/kg of medetomidine, 4.0 mg/kg of midazolam, and 5.0 mg/kg of butorphanol), as previously described. Pupils were dilated with Mydrin-P ophthalmic solution (1319810Q1053; Santen Pharmaceutical). The structural analysis of OCT data was performed using INSIGHT software (Phoenix Technology Group).

### Electroretinography (ERG)

ERGs were performed on the Rat/Mouse Ganzfeld with an LS-100 LED light-emitting device and PuREC PC-100 software (Mayo Corporation). Mice were dark-adapted overnight and anesthetized with a combination anesthetic. Pupils were dilated with tropicamide (Midorin-P, 1319810Q1053, Santen Pharmaceutical) under dim red light. Body temperature was maintained at 37 °C with a heating pad. A reference electrode was inserted intra-orally, a ground electrode was placed on the tail, and ERG responses were recorded from both eyes with gold electrodes placed on top of each cornea. Scotopic photoresponse was acquired under dark conditions with flashes of LED white light at 10 cd*s/m^2^, and the photopic photoresponse was recorded after light adaptation with background light (31.6 cd/m^2^) for 10 min; cone responses were acquired with flashes of white light at 30 cd*s/m^2^.

### Tissue immunostaining and TUNEL assay

Mice were sacrificed at the indicated age. Enucleated eyes were fixed with 4% Paraformaldehyde Phosphate Buffer Solution (4% PFA, Wako) at 4 °C overnight. After the removal of the lens, eyes were washed with PBS, immersed in 30% sucrose, and cryopreserved with O.C.T. (SAKURA). Cryosectioning was performed with a Leica CM1850 Cryostat. Retinal sections of 12 μm were mounted on MAS-GP slide glasses (Matsunami). Retinal sections were washed with PBS with 0.3% Triton X-100, incubated with Protein Block (Dako), and incubated with primary antibodies at 4 °C overnight. After washing with PBS with 0.05% Tween-20 (PBST), retinal sections were incubated with secondary antibodies at room temperature for 30 min. For ciliary protein immunostaining, retinal sections were incubated in Target retrieval solution (Dako) at 110 °C for 10 min before washing with PBS with 0.3% Triton X-100. Images were taken using an LSM 700 confocal microscope (Zeiss). Confocal images were captured for six areas per mouse to compare the number of retinal neurons between genotypes. The channels were separated depending on the antibodies used, and each image was automatically binarized using the same method. Cells were counted using the “Analyze Particle” method in ImageJ. To count cone and bipolar cells, the cell bodies indicated by the merged immunostaining of each antibody and DAPI were manually counted. To measure the relative cone cell position, the pedicle-to-cone cell body distance divided by ONL thickness was calculated from between 25 and 35 cells in each animal. The mean value of the relative cone cell position for each genotype was calculated from the mean value in each animal (n = 3 per genotype) and compared using a *t*-test. To quantify the CCDC181-immunostained volumes in photoreceptor primary cilia, 16–19 confocal images with a 53.35 μm width and 53.35 μm height obtained using a ×40 objective lens were stacked at an 8–10 μm depth in three areas. Each stacked image was processed using the 3D Objects Counter in ImageJ, and the obtained volume of each CCDC181-immunostained spot was averaged per mouse. To detect apoptotic cells in the mouse retina, ClickiT-Plus TUNEL Assay Kit (C10618, Thermo Fisher Scientific) was used according to manufacturer instructions. Cell counts in 320-μm widths were averaged from six sections for each mouse.

### Western blotting

Retinal tissue was isolated from sacrificed mice and lysed with TNE buffer (10 mM Tris, 1 mM EDTA, 150 mM NaCl, 1% NP-40) supplemented with cOmplete™ protease inhibitor cocktail (Roche diagnostics) and 1 mM PMSF (ALEXIS Biochemicals). Equal amounts of retinal lysates (4 μg/well) were denatured and separated by SDS-PAGE and transferred to polyvinylidene fluoride membranes using a Trans-Blot Turbo (Bio-Rad Laboratories). Blocking of membranes, incubation with primary antibodies, and incubation with secondary antibodies were performed using Can Get Signal (TOYOBO) following the manufacturer’s instructions.　After washing with PBST, membranes were incubated with SuperSignal West Femto Maximum Sensitivity Substrate (Pierce/Thermo Fisher Scientific), and signals were detected using a ChemiDoc XRS (Bio-Rad Laboratories). Protein expression levels were normalized by actin.

### Total retinal proteome analysis by mass spectrometry

To delineate the dynamics of the retinal proteome in the CCT2 mutant LCA model, retinal tissue was dissected from the right eyes of wild-type (n = 3) and homozygous R516H (n = 3) 4-w-old mice obtained from littermate pairs. All tissue samples were snap-frozen until processed for mass spectrometry (MS). For MS sample preparation, retinal tissues were lysed on ice with 50 µL of lysis buffer containing 20 mM HEPES, 150 mM NaCl, 1% NP-40, and 0.1% SDS. Protease inhibitor cocktail (Halt™) was added to lysis buffer just before lysate preparation. After centrifugation (4 °C, 15,000 rpm, 15 min), supernatant samples were processed as previously described^27^. Digested samples were pooled by group, desalted by tC18 SepPak (Waters), and then dried by an evaporator. Prepared proteomic samples were stored at −30 °C prior to MS analysis.

Retinal proteome analysis was performed via differential ion mobility MS on a FAIMS-Pro interface with Orbitrap Fusion Lumos Tribrid mass spectrometer (Thermo Fisher Scientific) as described previously, with minor alterations^28^. Briefly, the collision HCD was used and the compensation voltage (CV) sets were optimized for proteomic analysis as follows: CV set 1 (−40 V/−60 V/−80 V), CV set 2 (−50 V/−70 V/−90 V) and CV set 3 (−45 V/−55 V/−65 V). The acquired raw data were processed by Proteome Discoverer (version 2.4) with Mascot and Sequest HT engines to search proteins against the mouse proteome database (Swiss Prot version 2017_09). The mass tolerance for precursor and fragment masses were set to 10 ppm and 0.6 Da respectively. The carbamidomethylation of cysteine was included as a static modification and the oxidation of methionine was included as a dynamic modification. The false discovery rate (FDR) was set to 0.01. For label-free quantification (LFQ) analysis, three replicate analyses were performed in each CV set (total of 9 analyses per group) and processed for protein quantification.

Mouse WD40-repeat (WDR) proteins (n = 1398) were obtained from the public database WDSPdb 2.0 (http://www.wdspdb.com/wdsp/)^29^. The mouse WDR protein list is in Supplementary Data 2.

Gene ontology (GO) analysis was performed by PANTHER to identify differentially abundant proteins.

### Transmission electron microscopy

Mice were euthanized at 4 weeks. Eye cups were fixed with 2% glutaraldehyde, 2% PFA, and 0.1 M PBS buffer at 4 °C overnight. Dehydration, embedding, sectioning, and imaging were performed at Hanaichi Ultrastructure Research Institute (Aichi, Japan).

### Plasmid construction and immunoprecipitation

The construction of N-terminally Flag-tagged human CCT2 expression vectors (pEF-BOS-Flag-*CCT2* wild type, -T400P and -R516H) was described previously^6^. To generate the N-terminally Myc-tagged human CCDC181 expression vector, the coding sequence of CCDC181 (NM_001300969) was PCR-amplified from human retinal cDNA (Clontech, #639349) using primers 5′-TCGGTCGACCatgaatgaaaataaagatac-3′ and 5′-GATCGCGGCCGCtcagttataatgatcagtaaaac-3′. The amplified fragment was subcloned into the *SalI* and *NotI* sites of the pCMV-Myc vector (Clontech, #631604). The human *CCT2*, T400P mutant, and R516H mutant were co-transfected with human *CCDC181* into HEK293T cells with Lipofectamine 3000 reagent (Thermo Fisher Scientific) according to the manufacturer’s instructions. Cells were lysed with TNE buffer 3 days after transfection. Flag-tag Immunoprecipitation was performed using the Anti-DYKDDDDK tag Antibody Magnetic Beads (Fujifilm Wako) according to the manufacturer’s instructions. Immunoprecipitates and inputs were separated by SDS-PAGE as described for Western blotting. Membranes were immunoblotted with primary mouse anti-Myc (4A6, Millipore) or anti-Flag (M2, Sigma) antibodies and secondary TrueBlot anti-IgG HRP (eB270, Rockland Immunochemicals) antibody.

### Statistics and reproducibility

All quantitative analyses were performed on age-matched littermates of *Cct2* R516H knock-in and wild-type mice, or T400P/R516H compound heterozygous knock-in and wild-type mice. Statistical analyses were performed using R (v.4.0.5 with rstatix package v.0.7.1). Data are presented as mean ± SEM. The Student’s *t*-test was used to compare the two groups. To compare more than three groups, ANOVA and Tukey-Kramer post hoc test was used. Each group contained more than three mice. P values were represented by asterisks as follows; *: p < 0.05; **: p < 0.01; ***: p < 0.001. In the bar graphs of Fig. 4B, *: p < 0.01 (0.05/5) was used. Pup counts for each delivery were tested against theoretical Mendelian expectations by chi-square test.

### Supplementary information

Supplementary Information

Description of Additional Supplementary Files

Supplementary Data 1

Supplementary Data 2

Supplementary Data 3

Supplementary Data 4

## Data Availability

The LC/MS raw data and the identified protein result files for mouse retinal proteome have been deposited into a public open access proteomic database, the Japan Proteome Standard Repository/Database (jPOST), as follows: Mouse retina proteome (LFQ) for CCT2-LCA model in JPST002351. Uncropped blot images are in the Fig. S6. Antibodies used for immunostaining and western blotting are shown in Supplementary Data 3. Numerical source data for graphs are in Supplementary Data 4 file.
