# Supplementary material for: Compound heterozygous mutations in a mouse model of Leber congenital amaurosis reveal the role of CCT2 in photoreceptor maintenance
Source: Commun Biol. 2024 Jun 3;7:676. doi: 10.1038/s42003-024-06384-2 (PMC11148128; doi:10.1038/s42003-024-06384-2)
Supplement: Supplementary file 1 — Supplementary Information [file 42003_2024_6384_MOESM1_ESM.pdf]

Compound heterozygous mutations in a mouse model of Leber congenital amaurosis reveal the role of CCT2 in photoreceptor maintenance.

Akiko Suga[1], Yuriko Minegishi[2], Megumi Yamamoto[1], Koji Ueda[2], Takeshi Iwata[1*]

[1]Division of Molecular and Cellular Biology, National Institute of Sensory Organs, NHO Tokyo Medical Center, Tokyo, Japan

[2]Cancer Proteomics Group, Cancer Precision Medicine Center, Japanese Foundation for Cancer Research, Tokyo, Japan

These authors contributed equally: Akiko Suga and Yuriko Minegishi

*Corresponding author

Contact information: Takeshi Iwata, PhD, Division of Molecular and Cellular Biology Division, National Institute of Sensory Organs, NHO Tokyo Medical Center, 2-5-1, Higashigaoka Meguro-ku, Tokyo 152-8902 Japan, Phone: +81-3-3411-1026, Email: takeshi.iwata@kankakuki.jp

Supplementary figures and legends

Fig. S1: Generation of CCT2-T400P and -R516H mouse strains.

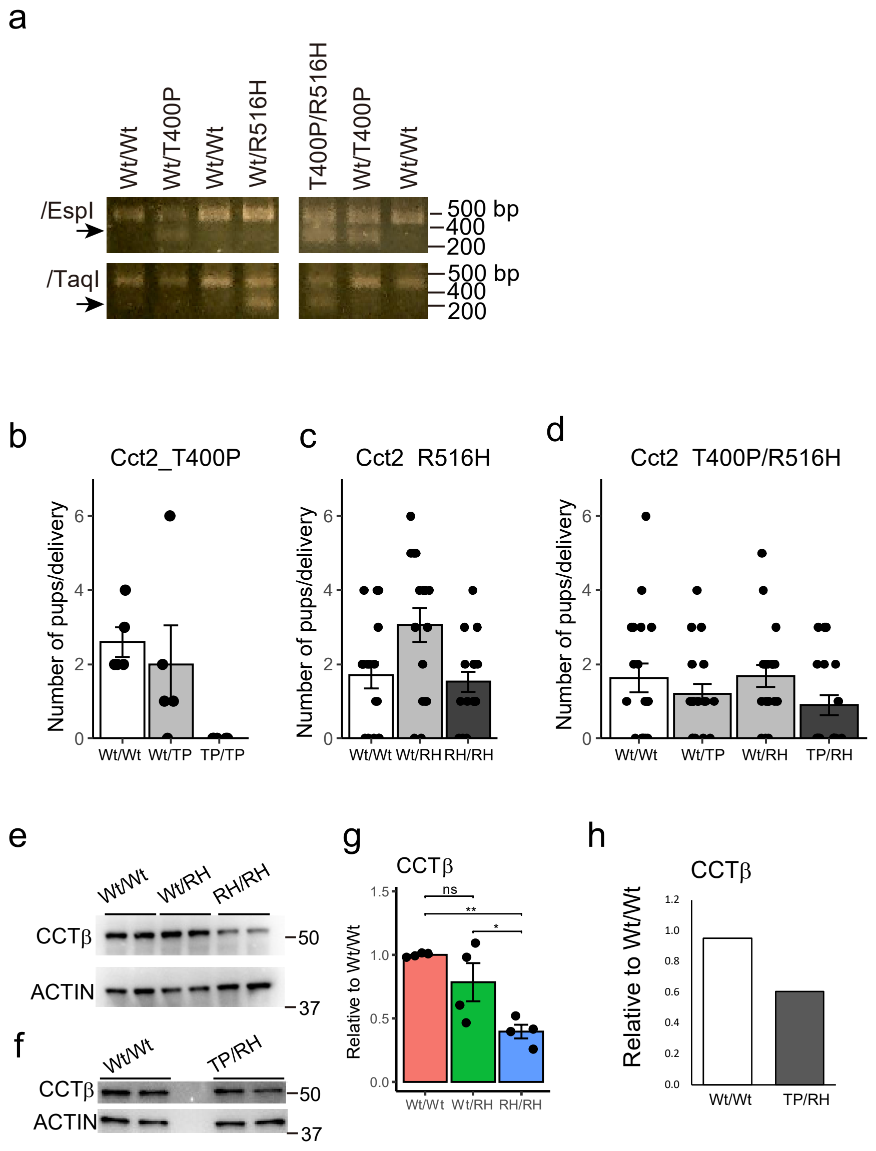

a Agarose gel electrophoresis showing the restriction enzyme-digested PCR products from wild-type (Wt/Wt), T400P heterozygote (Wt/T400P), R516H heterozygote (Wt/R516H), and T400P/R516H compound heterozygous mice. **b-d** The numbers of pups born after natural mating of heterozygous mice. Genotypes of the pups are indicated in X-axis. **b** Results of 5 cycles (T400P), **c** 17 cycles (R516H) and **d** 19 cycles (T400P/R516H) of mating. **e, f** Representative WB images of CCTβ protein in 4 w mouse retinas **e** and P14 mouse retinas **f**. **g, h** Quantification of the CCT2 protein amount at 4 w (**g**) and P14 (**h**).

Fig. S2: Expression patterns of *CCT2* mRNA in human and mouse retinas.

a Human retina

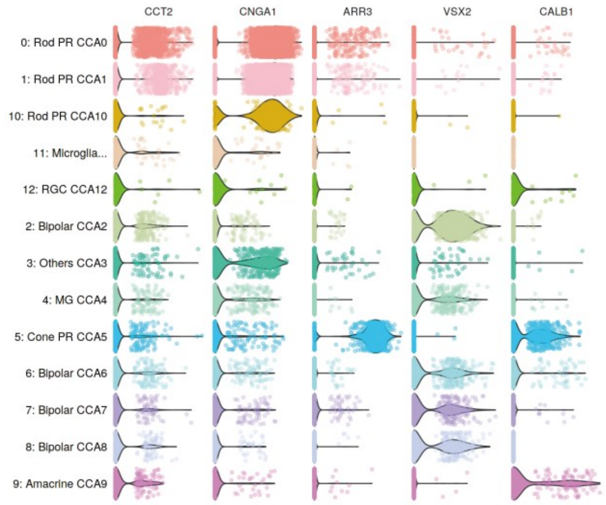

b Mouse retina

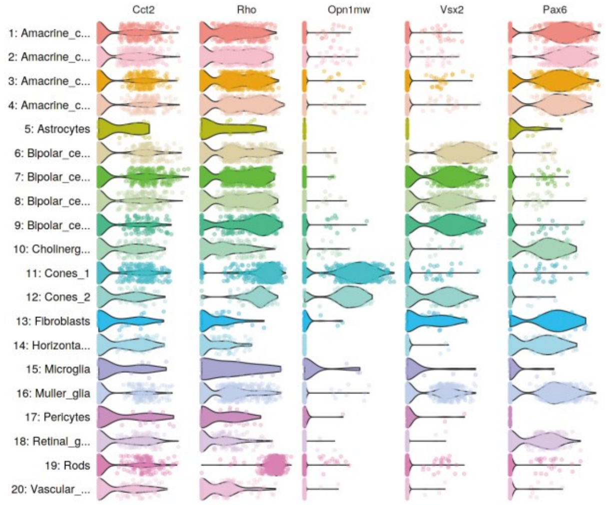

a Relative expression levels of *CCT2* and cell-type specific markers in human retina [1]. *CNGA1*: rod photoreceptors, *ARR3*: cone photoreceptors, *VSX2*: bipolar cells, and *CALB1* for amacrine cells. **b** Relative expression levels of *Cct2* and cell-type specific markers in mouse retina [2]. *Rho*: rod photoreceptors, *Opn1mw*: cone photoreceptors, *Vsx2*: bipolar cells, and *Pax6* for amacrine and retinal ganglion cells. Cell clusters are indicated vertically. Expression level in each cluster is horizontally displayed by violine plot. Previously-published single cell RNA-sequencing data for human and mouse retina are displayed by Spectacle [3].

Fig. S3: Phenotype of *Cct2* T400P heterozygous mouse retina.

a

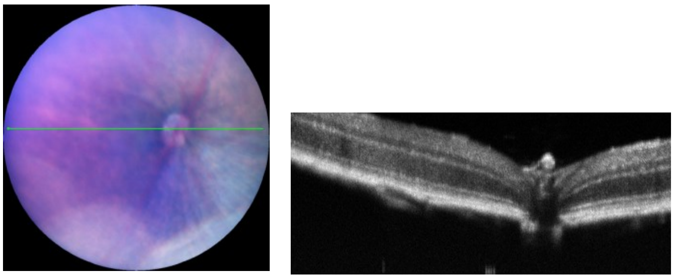

b

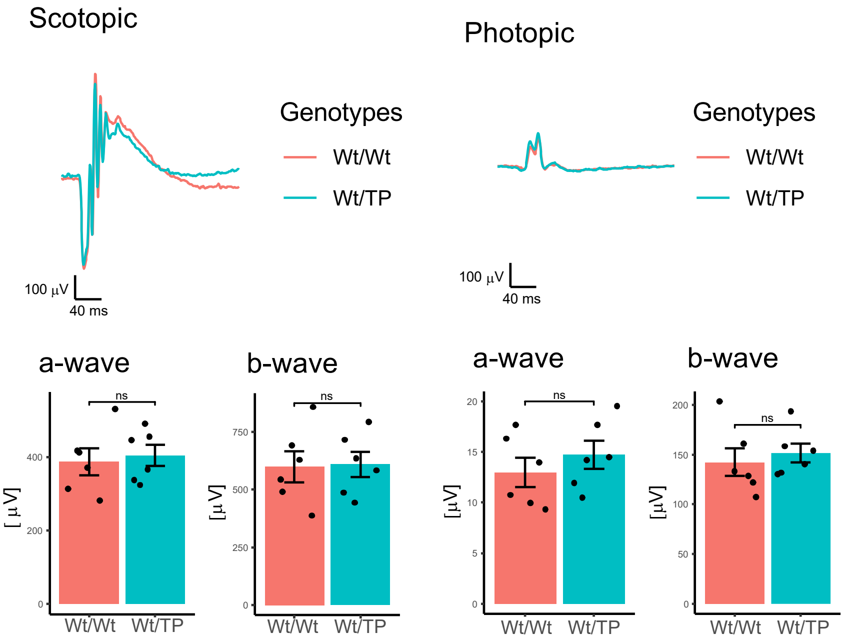

a Representative fundus image and OCT image of Wt/T400P retina at 43 w. **b** Scotopic and photopic responses of Wt/Wt and Wt/T400P (Wt/TP) mice at 43 w. Absolute value is indicated on y-axis. ns: not significant.

Fig. S4: CCDC181 enhanced tubulin acetylation *in vitro*.

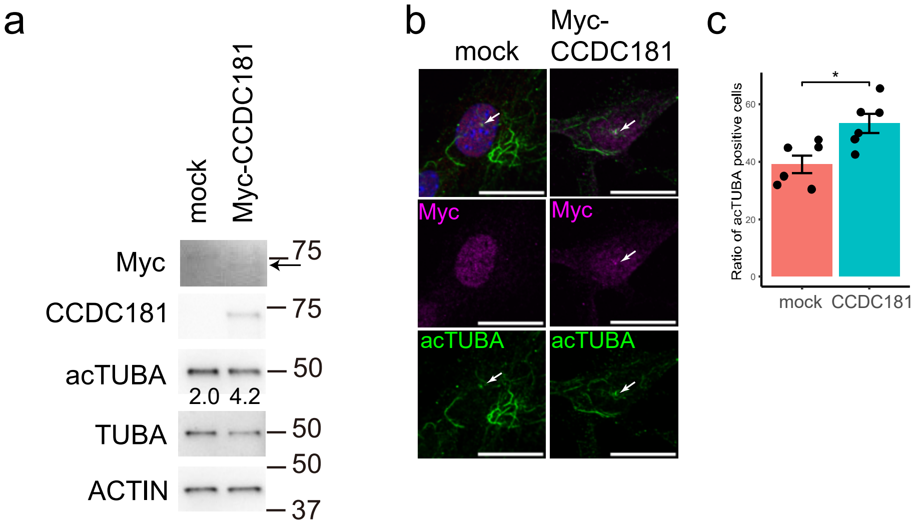

a Overexpression of Myc-tagged CCDC181 in NIH3T3 cells increased the ratio of acetylated alpha-tubulin (acTUBA) to alpha-tubulin (TUBA) after 3d of serum starvation. The ratio of acTUBA to TUBA is indicated below the acTUBA blotting. **b** Overexpressed Myc-tagged CCDC181 colocalized to acetylated tubulin in NIH3T3 cells after 3d of serum starvation. **c** Quantification of the number of acTUBA-positive NIH3T3 cells.

Fig. S5: Increase of insoluble GNAT1 in the R516H/R516H retina at 4 w.

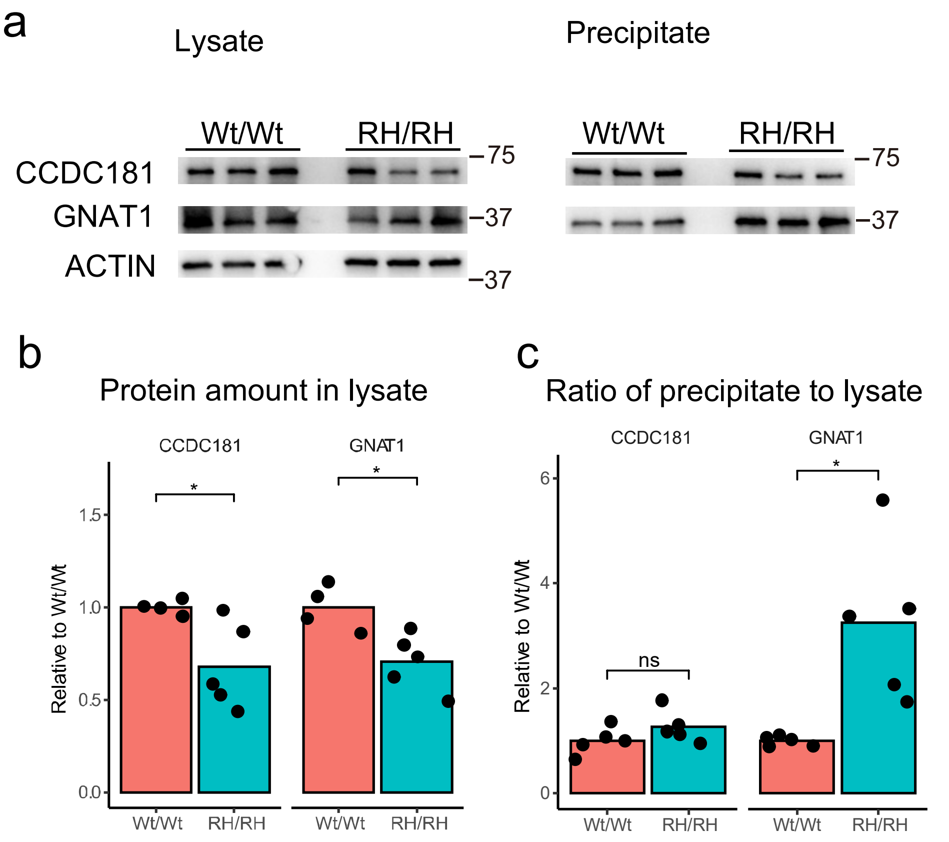

a Representative images of western blotting of retinal lysate from Wt/Wt and R516H/R516H (RH/RH). **b** Quantification of CCDC181 and GNAT1 proteins in the retinal lysate. Values were normalized by actin for each retina. Each dots indicate relative amount to the mean of Wt/Wt. **c** Soluble/insoluble ratios for CCDC181 and GNAT1 in retinas of indicated genotypes.

Fig. S6: Original blots for Figures 5a, 6a, 6e, and 6k.

Figure 5a

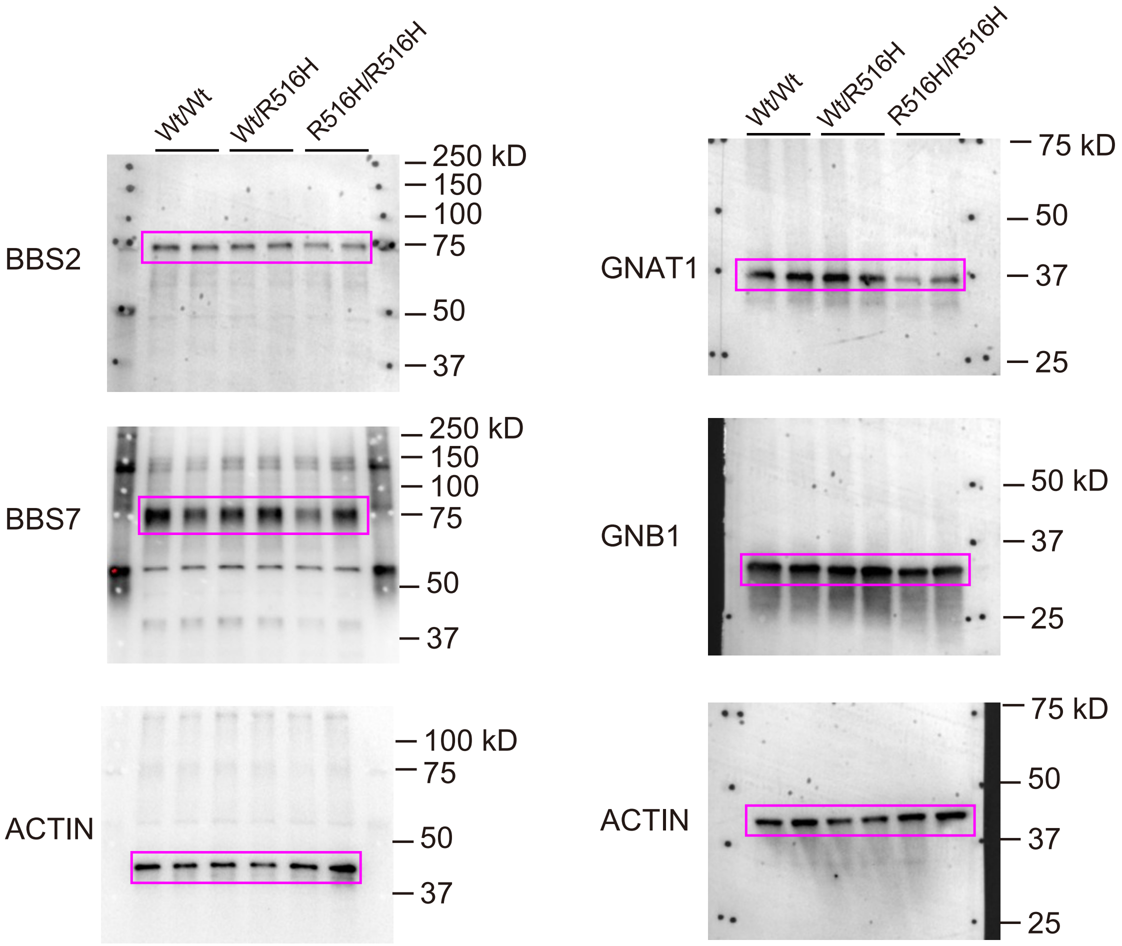

Fig. S6 (continued)

Figure 6a

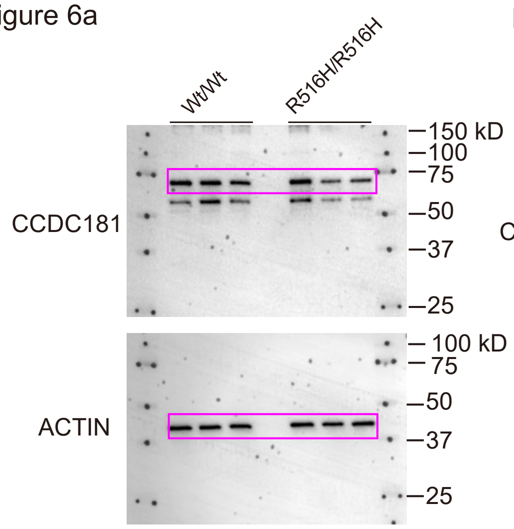

Figure 6e

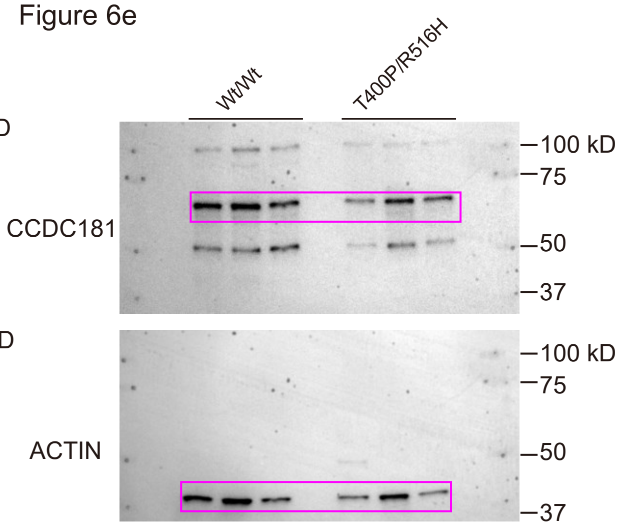

Figure 6k

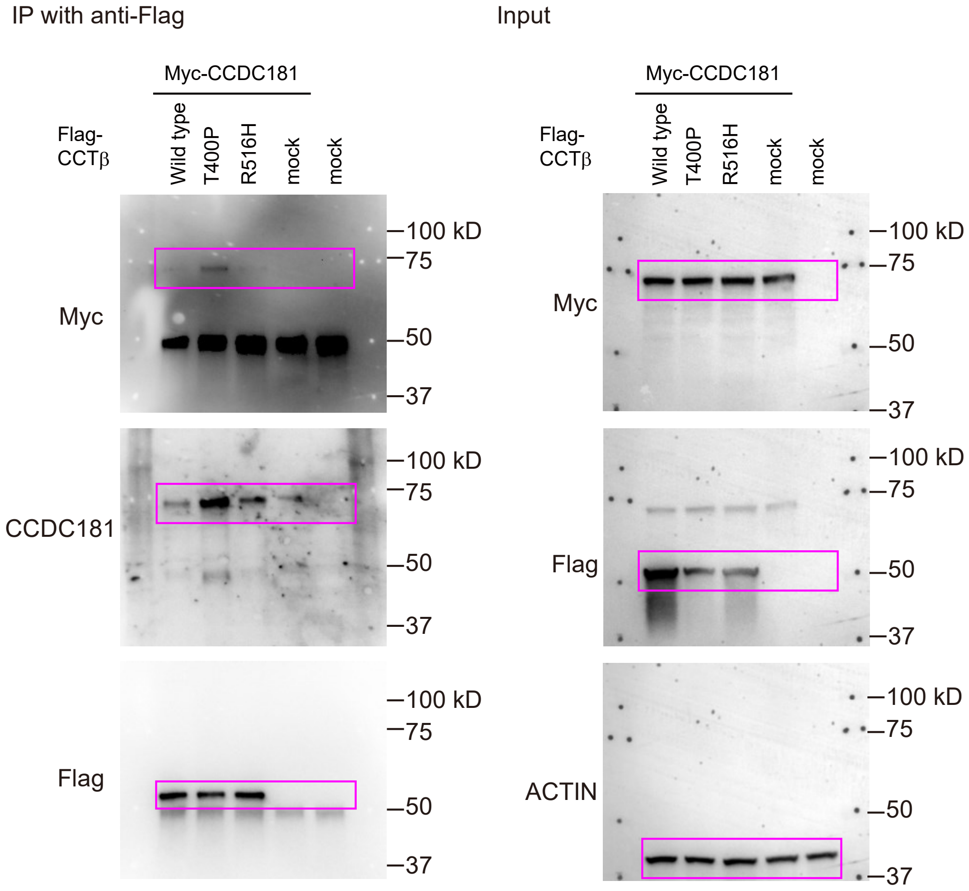

Figure S1a

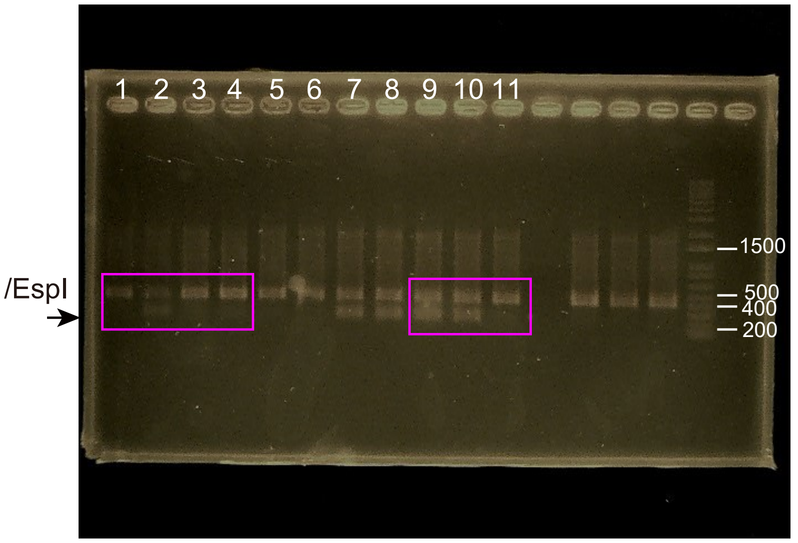

1: Wt/Wt
2: Wt/T400P
3: Wt/Wt
4: Wt/R516H
5: Wt/Wt
6: Wt/R516H
7: T400P/R516H
8: T400P/R516H
9: T400P/R516H
10: Wt/T400P
11: Wt/Wt

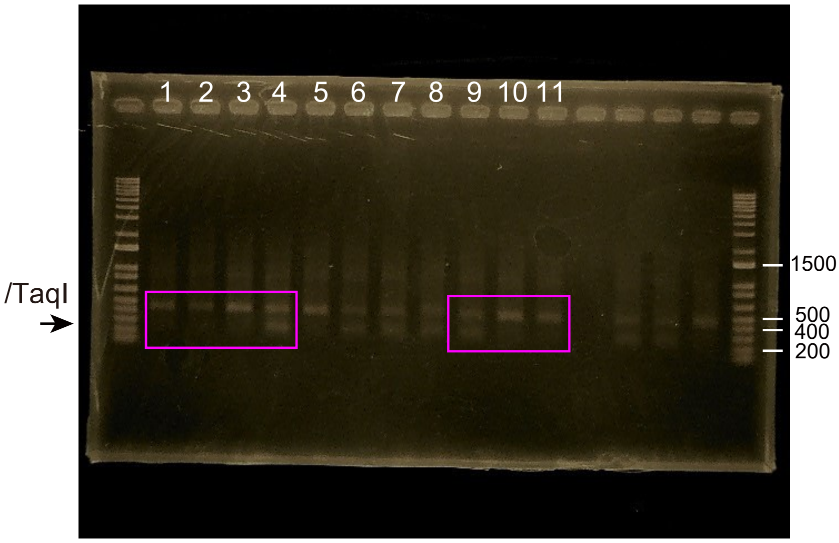

Supplementary references

1 Lukowski, S. W. *et al.* A single-cell transcriptome atlas of the adult human retina. *EMBO J* **38**, e100811 (2019). https://doi.org/10.15252/embj.2018100811

2 Macosko, E. Z. *et al.* Highly Parallel Genome-wide Expression Profiling of Individual Cells Using Nanoliter Droplets. *Cell* **161**, 1202-1214 (2015). https://doi.org/10.1016/j.cell.2015.05.002

3 Voigt, A. P. *et al.* Spectacle: An interactive resource for ocular single-cell RNA sequencing data analysis. *Exp Eye Res* **200**, 108204 (2020). https://doi.org/10.1016/j.exer.2020.108204
