# Supplementary material for: Compound heterozygous mutations in a mouse model of Leber congenital amaurosis reveal the role of CCT2 in photoreceptor maintenance
Source: Commun Biol. 2024 Jun 3;7:676. doi: 10.1038/s42003-024-06384-2 (PMC11148128; doi:10.1038/s42003-024-06384-2)
Supplement: Supplementary file 2 — Description of Additional Supplementary Files [file 42003_2024_6384_MOESM2_ESM.pdf]

# Description of Additional Supplementary Files

File name: Supplementary Data 1
Description: The full list of identified proteins by LFQ.

File name: Supplementary Data 2
Description: The mouse WDR protein list.

File name: Supplementary Data 3
Description: Antibodies used for this manuscript.

File name: Supplementary Data 4
Description: Numerical source data for graphs in the main manuscript.
